# Update of Pheochromocytoma Syndromes: Genetics, Biochemical Evaluation, and Imaging

**DOI:** 10.3389/fendo.2018.00515

**Published:** 2018-11-27

**Authors:** Rami Alrezk, Andres Suarez, Isabel Tena, Karel Pacak

**Affiliations:** ^1^Section on Medical Neuroendocrinology, Eunice Kennedy Shriver National Institute of Child Health and Human Development, National Institutes of Health, Bethesda, MD, United States; ^2^National Institute of Diabetes and Digestive and Kidney Diseases, National Institutes of Health, Bethesda, MD, United States; ^3^Cleveland Clinic, Adrenal Center, Endocrinology and Metabolism Institute, Cleveland, OH, United States; ^4^Provincial Hospital, Castellon, Spain

**Keywords:** pheochromocytoma, paraganglioma, genetics, biochemical classification, DOTATATE, PRRT

## Abstract

Pheochromocytomas and paragangliomas (PCCs/PGLs) are rare commonly benign neuroendocrine tumors that share pathology features and clinical behavior in many cases. While PCCs are chromaffin-derived tumors that arise within the adrenal medulla, PGLs are neural-crest-derived tumors that originate at the extraadrenal paraganglia. Pheochromocytoma-paraganglioma (PPGL) syndromes are rapidly evolving entities in endocrinology and oncology. Discoveries over the last decade have significantly improved our understanding of the disease. These include the finding of new hereditary forms of PPGL and their associated susceptibility genes. Additionally, the availability of new functional imaging tools and advances in targeted radionuclide therapy have improved diagnostic accuracy and provided us with new therapeutic options. In this review article, we present the most recent advances in this field and provide an update of the biochemical classification that further reflects our understanding of the disease.

## Introduction

According to the 2017 World Health Organization (WHO) classification of endocrine tumors, pheochromocytomas (PCCs) are tumors of the chromaffin cells that arise within the adrenal medulla (1), whereas paragangliomas (PGLs) are neural crest-derived neuroendocrine tumors (NETs) that can originate at any level of extra-adrenal paraganglia (from the skull base to the pelvic floor) (2). PCCs and PGLs arising from sympathetic paraganglia are characterized by catecholamine production whereas PGLs distributed along the parasympathetic chains of the head and neck (NHPGL) tend to be silent or pseudo-silent tumors (3–5).

The field of pheochromocytoma-paraganglioma (PPGL) is rapidly evolving. Many discoveries over the last decade have significantly improved our understanding of the disease. The identification of new hereditary forms of PPGL has led to the highest rate of germline susceptibility in cancer genetics at almost 40% (6, 7). In addition, other PPGL-related genes have been discovered and there are currently over 22 susceptibility genes identified. The genotype-phenotype correlation shown in many studies often dictates the clinical presentation of syndromic forms of the disease. These include associated biochemical profile, tumor location, malignant potential, aggressive clinical behavior, and overall prognosis. Furthermore, genetic identification provides valuable information for establishing a treatment plan and procures the rational for an appropriate guidance for follow-up surveillance.

**Figure 1:**
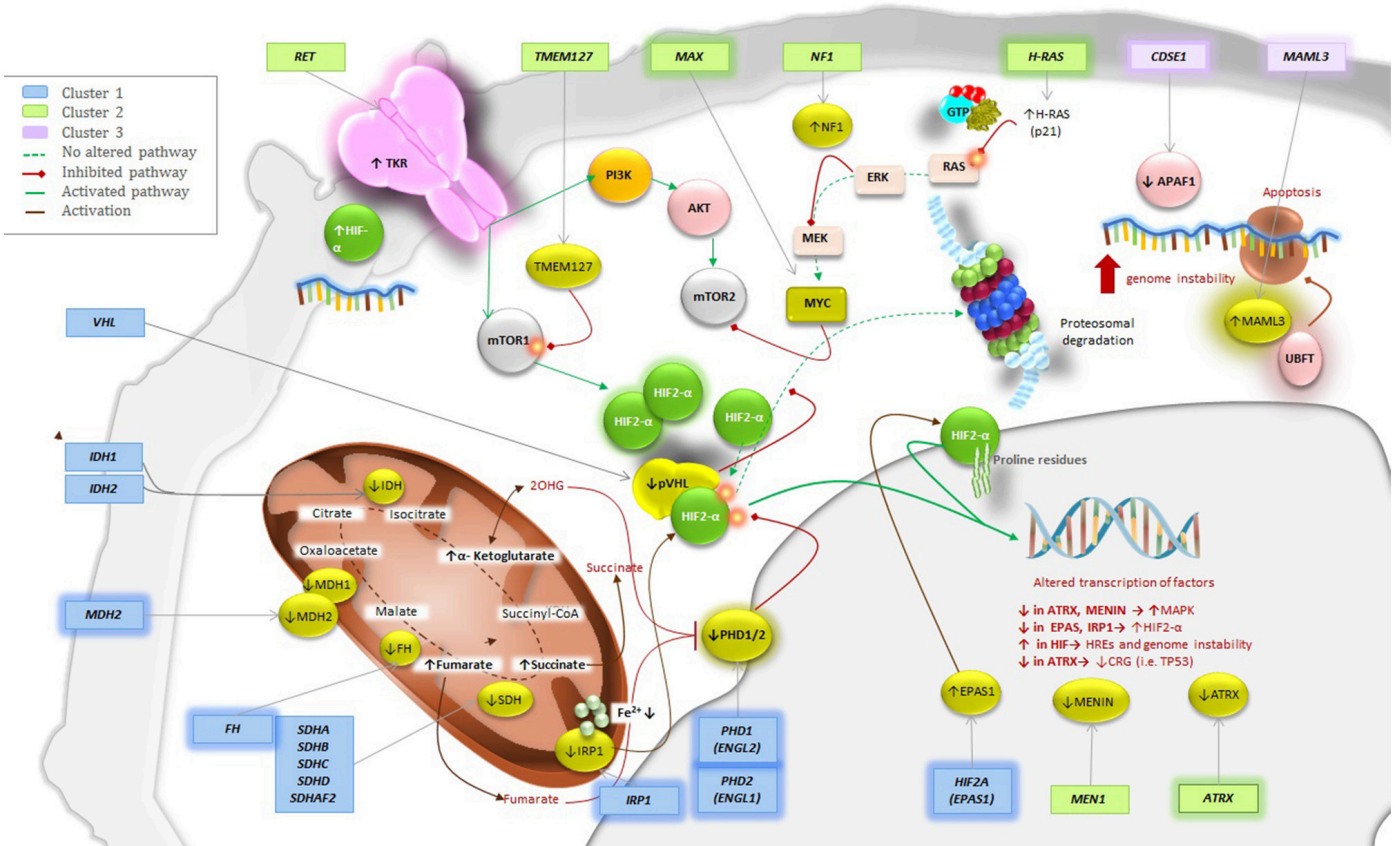
Genetics and molecular pathways for PPGLs Pathways for the New Genes; Placing the New into Perspective. Mutations of the highlighted genes have been discovered to play a role in the pathogenesis of PPGL. These genes can be classified in cluster 1, 2, or 3. Cluster 1 would be implemented with *FH, MDH2, PHD1 (EGLN2), EPAS1/HIF2A* and the most recently discovered *IRP1* that controls cellular iron metabolism and negatively regulates HIF2α mRNA translation. Cluster 2 would include *H-RAS* and *ATRX*, which belongs to the SWI/SNF family of chromatin remodeling proteins, as their upregulation will activate the RAS/RAF/ERK signaling pathway resulting in tumor formation. Finally, cluster 3 would be implemented with both *CSDE1* and *UBFT* fusion at *MAML3*. Alterations of any of this genes will result in increase of target genes involved in Wnt receptor and Hedgehog signaling pathways.

The involvement of the Krebs cycle and the respiratory chain, mainly represented by the involvement of succinate dehydrogenase (SDH) in the etiology of PPGL, is perhaps the most important discovery in this area. Mutations of genes that encode any of the subunits A, B, C, or D or the complex assembly factor 2 (AF2) account for a group of overlapping yet distinct hereditary syndromes termed SDHx. These forms are considered the most common of all hereditary PPGL syndromes accounting for ~30% of them (6, 7). Over the past 6 years, ten new genes have been discovered. These new genes include hypoxia-inducible factor 2 alpha (*HIF2A)*—also known as endothelial PAS domain-containing protein 1 (*EPAS1*) (8, 9), fumarate hydratase (*FH)* (10), HRAS proto-oncogene (*H-RAS)* (11), prolyl hydroxylase 1 (*PHD1)*—also known as egl nine homolog 2 (*EGLN2*) (12), malate dehydrogenase 2 (*MDH2)* (13), chromatin remodeler ATRX (*ATRX)* (14, 15), H3 histone family member 3A (*H3F3A*) (15), cold-shock domain containing E1 (*CSDE1)* (15), coactivator 3 mastermind-like (*MAML3*) fusion genes [upstream binding transcription factor, RNA polymerase I] *(UBTF)–MAML3* (15), and iron regulatory protein 1 (*IRP1)* (16). Multiplicity regarding mosaicism underlines also in some syndromes (8, 9, 17, 18). Each of these genes mutations affects a specific metabolic pathway. As a result, The Cancer Genomic Atlas (TCGA) group proposed a comprehensive system to classify PPGL-susceptibility genes into a molecular level. Based on genomic analysis, the system divides PPGL-related genes into four major clusters: a pseudohypoxia subtype (subdivided into tricarboxylic acid (TCA) cycle-dependent and *VHL/EPAS1*-dependent), a kinase-signaling subtype, a Wnt signaling subtype, and a cortical admixture subtype (Figure 1). These integrative efforts show that PPGL can be driven either by germline, somatic, or fusion genes mutations in 27, 39, and 7% of the cases, respectively (15).

## Genetics

Advances in genetics over the last decade have allowed the implementation of whole exome sequencing (WES) in developed countries as the new standard screening tool for genetic testing in patients with a suspected hereditary form of PPGL. However, the application of this technology is limited to specialized centers in developing countries. When available, the cost of testing is often a barrier for wide implementations of WES. Immunohistochemistry (IHC) is an alternative method in these cases, given its affordability and feasibility. It is used as screening tool where negative IHC for a gene can serve as an indirect indicator of the presence of a mutation in the gene of interest. This technique is especially useful in the context of suspected *SDHx* mutations (19–22). False-positive or false-negative results (19–22) are not uncommon, therefore, IHC should be interpreted with caution.

**Figure 2:**
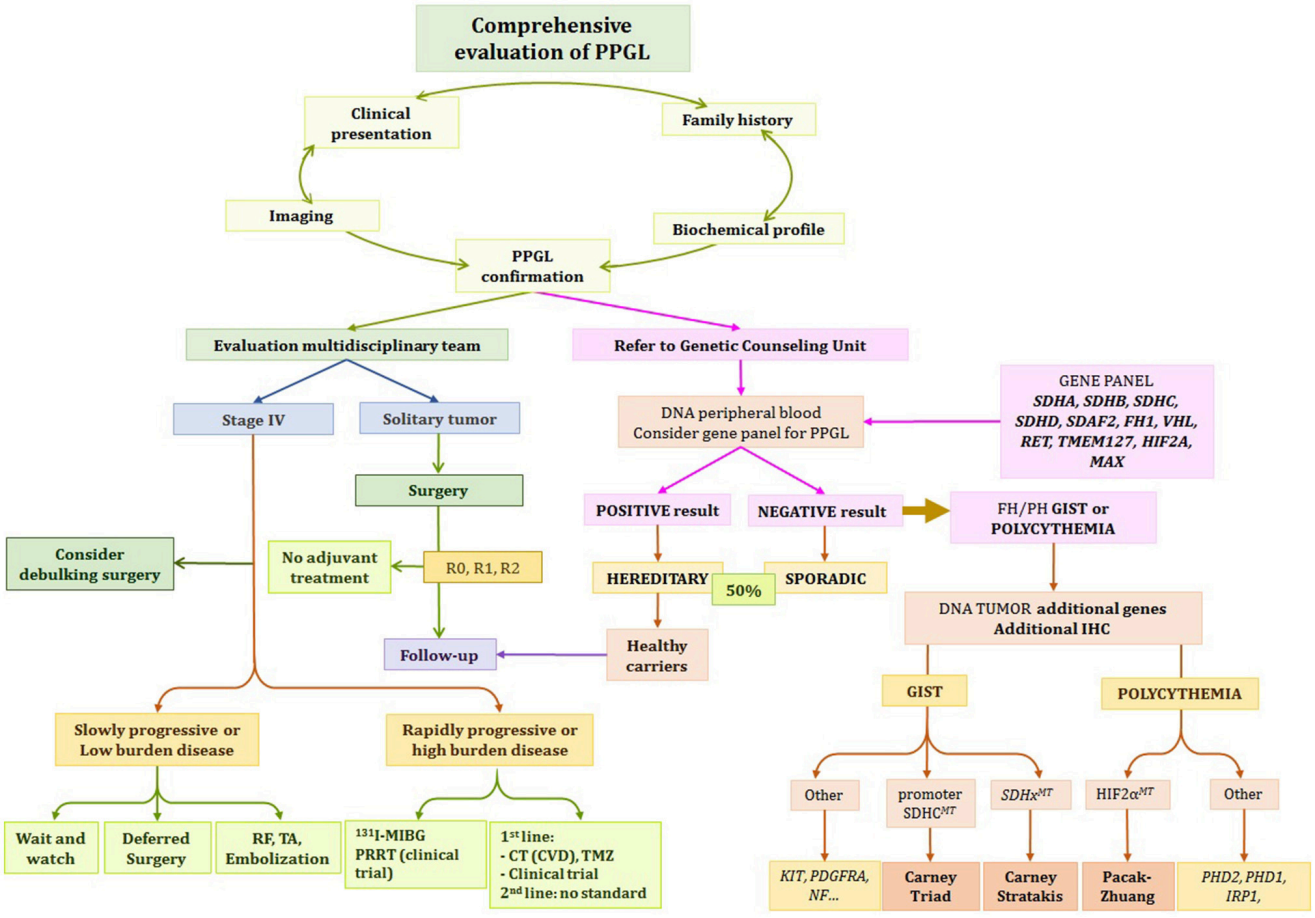
Proposed Algorithm for Evaluation and Management of PPGL Patient. *Depending on clinical status, growth acceleration, symptoms, and genetic status, the options include: observation, systemic therapy, chemotherapy, PRRT, ^131^I-MIBG, or referral to clinical trials. R0, microscopically margin-negative resection; R1, microscopically margin-positive resection; R2, macroscopically margin-positive resection; stage IV, metastatic disease (1).

Integrative patient evaluation is essential as the clinical presentation, along with radiological and biochemical profile will guide clinicians toward the correct genetic diagnosis. This holds true when WES is not readily available. In these cases screening for specific gene or gene panels for a subgroup of susceptibility genes can be an alternative option. Therefore, we emphasize the importance of phenotype profile recognition (Table 1, Figure 2).

**Table 1:** Genetics and clinical profile for the newly discovered forms of PPGLs.

**Gene**	**Syndrome**	**Biochemical profile**	**Date of discovery**	**Gene role**	**Clinical presentation**	**Gene type**	**Cluster**	**Inheritance**	**References**
*FH*	HLRCC	Noradrenergic	2012	TSG; encodes FH that catalyzes the reversible hydration of fumarate to l-malate in the TCA cycle. Increase in fumarate leads to stabilization of HIF	Multifocal, metastatic, associated with RCC and leiomyomatosis	G	1	AD	(10, 23–25)
*HIF2A or EPAS1*	Pacak-Zhuang	Noradrenergic	2012	Oncogene; encodes EPAS1; transcription factor related to oxygen level responses and activated in hypoxic conditions	Triad of PPGLs, polycythemia, and somatostatinoma. Ocular abnormalities occur in 70%	S/M	1	N/A	(8, 9, 24, 26)
*H-RAS*		Adrenergic	2013	Proto-oncogene; encodes H-RAS (P21), that once bound to GTP, activates the RAS/RAF/ERK signaling pathway leading to cell proliferation	Unilateral PCC, sporadic, benign	S	2	N/A	(11, 27)
*H3F3A*		Unknown	2013	Encodes the histone H3.3 protein. responsible for chromatin regulation	Giant cell tumors of the bones (GCT), PCCs, bladder and periaortic PPGL	S	*	N/A	(28)
*EGLN2(PHD1)*		Noradrenergic	2015	TSG; encodes PHD1, enzyme which in normal oxygen conditions, hydroxylates specific proline residues of the HIF-α subunits for posterior degradation in the proteasome	Polycythemia associated with recurrent PPGLs, and normal or mild elevated EPO	G	1	**	(12)
*MDH2*		Noradrenergic	2015	TSG; encodes MDH2 that catalyzes the reversible oxidation of malate to oxaloacetate in the TCA cycle.Increase in malate, fumarate and succinate leads to stabilization of HIF	Multiple PGLs	G	1	AD	(13)
*ATRX*	ATRX	Noradrenergic	2015	Encodes the transcriptional regulator ATRX	Clinically more aggressive and metastatic PGL	S	*	N/A	(29)
*CSDE1*		Adrenergic	2016	Tumor suppressor gene. Involved in normal development through messenger RNA stability internal initiation of translation, and cell-type-specific apoptosis.	Sporadic, metastatic, recurrent PPGL	S	*	N/A	(15)
*UBTF-MAML3* fusion		Adrenergic	2016	Oncogene. In PPGLs, unique hypomethylation profile mRNA overexpression of target gene involved in Wnt receptor and Hedgehog signaling pathways	Sporadic, recurrent PGL. New prognostic factor of poor outcome	F	3	N/A	(15)
*IRP1*	IRP1	Noradrenergic	2017	TSG; encodes IRP1, that controls cellular iron metabolism and negatively regulates HIF2α mRNA translation under iron-deficient conditions. Deficiency of IRP1 protein increases HIF2α	Sporadic, adrenal PCC	S	1	N/A	(30)

*S, somatic; G, germline; M, mosaicism; F, fusion; RCC, renal cell carcinoma; HLRCC, hereditary leiomyomatosis and renal cell cancer; PCC, pheochromocytoma; PPGL, pheochromocytoma-paraganglioma; TSG, tumor suppressor gene; GCT, giant cell tumor*.

*N/A, Not Applicable in the setting of somatic mutations*.

*AD, Autosomal Dominant*.

* *Not classified by clusters*,

** *Unknown*.

## Cluster 1/Pseudohypoxia subtype

### 1A. TCA cycle-related

#### FH:

*FH* is a tumor suppressor gene in the TCA cycle that encodes FH enzyme, which converts fumarate into malate (31). Deficiency in FH results in accumulation of the precursor metabolite fumarate, which shares structural similarities with succinate, leading to prolyl-hydroxylase (PHD) inactivation and HIF stabilization (32, 33). *FH* has an autosomal dominant inheritance with variable expressivity. Patients typically present with multiple cutaneous leiomyomatosis (MCUL). Leiomyomas are smooth muscle tumors that arise from the skin and uterus in these patients. When associated with renal cell cancer, this syndrome is referred to as hereditary leiomyomatosis and renal cell cancer (HLRCC) also known as Reed's syndrome (23). PPGL is a rare second manifestation of the syndrome and tends to be malignant and/or multiple and present with a predominant noradrenergic profile. To date, only few cases have been reported and both pathogenic germline and somatic mutations have been described (10, 34). Age of presentation varies widely from as young as 6- to 70-years-old. More recently, a study of a cohort reported by the National Institutes of Health (NIH) revealed co-secreting dopamine (DA) tumors in some *FH* mutated patients (24).

### 1B. *VHL/EPAS1*-related

#### EPAS1/HIF2A:

*EPAS1* (*HIF2A*) is an oncogene that encodes the EPAS1; a transcription factor related to oxygen level responses (35). In 2012, Zhuang et al. were the first to identify a gain-of-function somatic mutation in exon 12 of *EPAS1* (G1588A and C1589T) in patients with PPGLs. These mutations caused defects on the proline residues at the hydroxylation site of HIF-2α leading to its reduced degradation and stabilization (8, 9). Since then, other cases of PPGL with polycythemia have been reported. Among them, somatic mutations of *HIF2A* at exon 12 are responsible in most cases of the disease, and only one case was caused by germline mutation of *HIF2A* at exon 9 (36). Furthermore, both germline mosaicism/somatic mutations have been described in additional studies (18, 36). Somatic *HIF2A*-related PPGL affects predominantly females and patients typically presents with PPGL, somatostatinoma and polycythemia (Pacak-Zhuang syndrome) (8). However, penetrance can vary from patient to patient and incomplete forms of the disease have been reported. In particular, the presence of somatostatinoma has not been described in males yet. PPGLs in these patients are often multiple or recurrent with elevated norepinephrine (NE), normetanephrine (NMN), DA, and erythropoietin (EPO) and once third of the patients present with metastatic disease (37). Also, ~70% of the patients have been found to have ocular abnormalities with bilateral dilated capillaries and fibrosis overlying the optic disc being the most common findings. Therefore, an early referral to an ophthalmologist acquainted with the retinal findings of this syndrome is strongly recommended (38). Surgery of the PPGL, often results in an improvement of *HIF2A*-induced polycythemia but treatment of the disease still requires intermittent phlebotomies, and control blood pressure to prevent complications resulting from polycythemia.

### Cluster 2/Kinase signaling group

#### H-RAS:

Located on chromosome 11p.15.5, *H-RAS* is a proto-oncogene that, encodes H-RAS factor (also known as transforming protein p21). H-RAS, once bound to guanosine-5'-triphosphate (GTP), activates the RAS/RAF/ERK signaling pathway that will ultimately result in cell proliferation (27). Pathogenic mutations in *H-RAS* were firstly identified in 2013. WES of tumor DNA in four cases with phenotype suggesting an underlying pathogenic genetic variant revealed the presence of 2 hotspot mutations in *H-RAS* (G13R and Q61K) in two of them. In all four samples, known susceptibility genes had been previously excluded (11). All four cases were male patients with unilateral, sporadic/benign tumors (three PCCs and one abdominal PGL), along with elevation in plasma catecholamines. Further validation in a cohort of 58 additional samples obtained showed an accumulated frequency of 6.9% (4/58) of missense somatic mutations in *H-RAS*: G13R (*n* = 1), Q61K (*n* = 1), and Q61R (*n* = 2) (11).

### Cluster 3/*WNT* signaling group

#### CSDE1:

Formerly known as upstream of N-ras (*UNR*), *CSDE1* is a tumor suppressor gene located at chromosome 1p13.2 that encodes CSDE1 factor, which is mainly involved in development and has several functions including messenger RNA stability, internal initiation of translation, cell-type-specific apoptosis and neuronal differentiation (39, 40). The association of *CSDE1* to PPGL was recently described by TCGA group in a cohort study of 176 PPGL patients, in which four were found to have a somatic mutation— two frameshift, and two splice-site—, in *CSDE1* (15). Underexpression of CDSE1 has been reported in several tumors (41). Mutations in this gene result in downregulation of the apoptosis protease activator protein 1 (APAF1), which controls apoptosis in PCC cells under normal conditions (42, 43). Clinically, patients presented with sporadic cases, and some of them with recurrence or metastatic disease proposing a more aggressive form of PPGLs (15).

### *UBTF-MAML3* fusion:

*MAML3* (4q.31.1) is an oncogene that had been previously associated with other tumor types (44, 45). On the other hand, *UBTF* (17q21.31), encodes UBTF required for the expression of rRNA subunits (46). The association of the fusion with PPGL was identified by TCGA group in 2017 when analysis from RNA sequencing of the same cohort mentioned above revealed that ten tumors (eight primary, and two primary-metastatic) were positive for a *MAML3* fusion gene. Patients presented with a unique and expansive hypomethylation profile that was correlated with mRNA overexpression of target genes involved in developmental pathways, such as Wnt receptor signaling and Hedgehog signaling, that were significantly overexpressed including miR-375, β-catenin, DVL3, and GSK3 (15). MAML3-related PPGLs had the highest Ki-67 index expression, and some patients presented with aggressive disease (15). Finding *UBTF-MAML3* fusion predicts a poor prognosis, as compared with other syndromic forms of PPGL (15).

## Proposed classification for new genes according to TCGA

Three additional genes were not included in the molecular classification done by TCGA (15). However, based on their signaling pathways, we believe that all three should be included as part of the cluster 1 or pseudohypoxia signaling group for future updates. While *MDH2* is a part of the TCA cycle, both *PHD1* (*EGLN2*) and *IRP1* belong to the *VHL/EPAS1*-related subtype (Figure 1) and have been very recently described.

### MDH2:

*MDH2* encodes the MDH2 enzyme, which catalyzes the reversible oxidation of malate to oxaloacetate in the TCA cycle. Deficiency of MDH2 has shown to lead to the accumulation of malate, fumarate, and succinate on Drosophila models (47). Thus, *MDH2* should be classified as a cluster 1-TCA cycle-related gene. In 2015, Cascon et al. were the first to report a case with a germline mutation in *MDH2*: a male patient diagnosed with multiple PGLs (13). Later, five asymptomatic relatives were tested, and two were found to be positive. Subsequent updates showed elevation of NE and the presence of a hypermethylator phenotype similar to *SDHx-*related PPGLs (48). Interestingly, no malate accumulation was found on tumor cells, but a high fumarate/succinate ratio was observed (13).

### PHD1 (EGLN2):

*PHD1* (*EGLN2*) encodes PHD1 enzyme, which in normal oxygen conditions hydroxylates specific proline residues of the HIF-α subunits for their subsequent degradation by proteasome. Deficiency of this enzyme prevents degradation of HIF-α and resulting in HIF stabilization leading to a global activation of signaling pathways that lead to tumorigenesis (49). *PHD1* should therefore be classified as cluster 1-*VHL/EPAS1*-related gene. In 2008, Ladroue et al. were the first to describe the association of mutations in *PHD2* with polycythemia and PPGLs (50). Seven years later, Yang et al. reported the first mutation in *PHD1* (*EGLN2*), found to be associated with PPGL, along with an additional case with *PHD2* (*EGLN1*) germline mutation. These two patients presented with multiple recurrent PPGL, polycythemia with normal or mildly elevated EPO, and were negative for *HIF2A* mutation. Both patients presented with catecholamine related symptoms including: headaches, episodic chest pain, anxiety, and hypertension. Both cases revealed a noradrenergic profile. In the patient with the mutation in *PHD1 (EGLN2)*, sensitivity of erythroid progenitors to EPO and erythropoietin receptor (EpoR) activity were inappropriately increased and resulted in polycythemia with no or mild increase in EPO levels with increased EpoR expression (12).

### IRP1:

*IRP1* is a tumor suppression gene that encodes IRP1, a bi-functional protein that controls cellular iron metabolism and negatively regulates HIF2α mRNA translation under iron-deficient conditions (51, 52). Thus, deficiency of IRP1 increases HIF2α by dissociating sequences of HIF2α mRNA from iron-responsive element and suppressing protein translation. Activation of HIF-2α leads to an increase of transcription of EPO and EpoR. Recently, *IRP1* association with PCC was reported by Pang et al. in a patient with a medical history of polycythemia vera with a proven *JAK2* mutation, hypertension, diaphoresis, and abdominal pain that led to the diagnosis of PCC years later. An investigational gene panel consisting of 54 tumor-associated genes was negative for patient's peripheral blood DNA. Subsequent tumor DNA sequencing revealed a somatic, loss of function mutation in *IRP1* located on exon 3 splicing site (16).

### Driver mutation gene with unknown classification

#### H3F3A

Located on chromosome 1, *H3F3A* encodes the histone H3.3 protein (53). Histones are responsible for nucleosome formation, and as a chromatin regulator, mutations of this gene will affect DNA methylation, chromatin remodeling, or nucleosome positioning (54). Defects in *H3F3A* have been linked with diffuse intrinsic pontine glioma (DIPG) (55, 56), chondroblastoma, and giant cell tumor of the bone (GCT) (57). Association with PPGL was initially reported in 2013 as a case report (58). Three years later, in 2016, Toledo et al. analyzed 43 samples of 41 patients using exome or transcriptome sequencing and detected a postzygotic *H3F3A* mutation in three tumors from one patient with a history of recurrent GCT. This patient presented with bilateral PCCs and developed bladder and several periaortic PGLs later. Family history of PPGL was absent. Further analysis showed that this mutation was identical to an oncogenic driver of sporadic GCT (c.103 G > T, p.G34W) (57, 58). Furthermore, additional variants in other chromatin remodeling genes (*KMT2B, EZH2, SETD2, ATRX, JMJD1C, KDM2B*) were reported in this study (28). Additionally, two kinase receptor-encoding genes (*MERTK, MET*) were found (28). Also, the investigators detected a somatic mutation in the main hotspot residue of the fibroblast growth factor receptor 1 gene (28), which is known to play a role in other cancers, such as glioblastomas (59). Further studies are needed to clarify the role of these genes in the pathogenesis of PPGL.

## Disease modifying gene

### ATRX

Located on the X chromosome, *ATRX* encodes the transcriptional regulator ATRX, which belongs to the SWI/SNF family of chromatin remodeling proteins. ATRX plays a role in the histone deposition in telomeres, chromosome segregation in the cell cycle and transcription regulation (60, 61, 62). Germline mutations in *ATRX* have been reported as a cause of X-linked alpha thalassemia mental retardation syndrome (ATRX syndrome) (63). In 2015, using WES, Fishbein et al. reported somatic *ATRX* mutations in two *SDHB-*related frozen tumors. They found somatic *ATRX* mutations in 12.6% of the samples, along with an alternative lengthening of telomeres seen on fluorescence *in situ* hybridization (FISH) and presenting with more aggressive disease (29). Later, the first case of an *ATRX* driver mutation was reported in a patient with a metastatic composite PCC-PGL, clinically with anemia, weight loss, and hepatic metastases. WES showed a somatic loss of function on the *ATRX* gene, along with downregulation of genes involved in the neuronal development and homeostasis (*NLGN4, CD99*, and *CSF2RA*) and upregulation of *Drosha* gene related with RNA processing and alternative lengthening of telomeres (14). Somatic mutations have been reported in association with co-existing mutations in the isocitrate dehydrogenase 1 and 2 (*IDH 1/2*) genes in both adult and pediatric patients with astrocytic tumors (64). In addition, ATRX may play a driver mutation role for sporadic PPGL (14) and truncated ATRX could potentially play a synergistic role with *SDHx* in tumor initiation and might be a predisposition for a more aggressive disease (15).

### Biochemical evaluation

Biochemical evaluation has made tremendous strides forward since the 1950's when colorimetric assays were first implemented, and later replaced with the more accurate high-performance liquid chromatography (HPLC) in the 1980s. Currently, liquid chromatography tandem-mass spectrometry (LC-MS) has become the new gold standard due to its accuracy and reproducibility. A value three times the upper range of normal is a positive result. However, some patients present with pseudo-silent PPGL and high tumor burden resulting in a late diagnosis. Therefore, any values above the normal range should be carefully considered as positive in the setting of prospective screening in hereditary forms of the disease, given early proactive surveillance has made pre-detection of tumors very possible.

Besides screening and diagnoses, biochemical phenotype of the tumor is a useful tool for PPGL syndromic assessment. PPGLs can be classified according to their biochemical profile. This classification allows the establishment of an algorithm and addresses specific causative genes. Here, we present an update of the different PPGL-related biochemical phenotypes.

A- Truly biochemically silent phenotype: Often associated with *SDHx* syndromes, truly silent PPGLs are mostly located in the head and neck area (HNPGL). Among HNPGL, carotid body tumors are the most frequent (65), followed by glomus vagale, jugulotympanic, and laryngeal PGLs.

B- Biochemically pseudo-silent phenotype: In this category, PPGLs present with levels of catecholamines and metanephrines that can be “normal” or “near-normal” in a misleading way. This category should be distinguished from group A since PPGLs in this category are indeed catecholamine-producing tumors. However, detection of elevated metanephrine and normetanephrine falls below the limit of detection due to either low tumor burden or catecholamine production fluctuations. This usually happen in patients with very small (less than 5–7 mm) PPGLs.

C- Noradrenergic phenotype: Characterized by increased levels of NE/NMN (66, 67), noradrenergic PPGLs are commonly located outside the adrenals (66, 67), NE acts on both α (1 and 2) and β (1, 2, and 3) adrenoreceptors with predominant effect on α. These patients present less frequently with paroxysmal symptomatology. Sustained hypertension and tachycardia are the most common symptoms. However, hypertensive crisis, myocardial infarction, lethal tachyarrhythmia, and acute intramural hemorrhage have been reported (68, 69). This phenotype is commonly seen in the cluster 1/ pseudohypoxia group (15), including both *VHL* and *SDHx* mutations (70).

D- Adrenergic phenotype: These PPGLs are characterized by either purely elevated epinephrine (E)/metanephrine (MN) (66), or in both E/MN and NE/NMN. This phenotype can be accurately identified when the plasma level of free MN is greater than 10% of the sum of NMN and MN (66). Adrenergic PPGLs are often located in the adrenal gland (67). Epinephrine represents a higher level of cellular differentiation of adrenal PPGL when compared with tumors derived from paraganglia (71, 72). Epinephrine activates α-1 and α-2 adrenoceptors with a higher affinity compared to NE (73) but also affects β-2 adrenoceptors. Paroxysmal symptomatology due to a rapid metabolism (74) has been related to the concomitant use of medications like histamine, tricyclic antidepressants (TCA), anesthetics, and tyramine-rich food (75). In many cases these patients are found to have hyperglycemia and hyperlipidemia secondary to the stimulus of lipolysis, glycogenolysis, and gluconeogenesis (68). This phenotype is commonly seen in the cluster 2/kinase signaling group (15).

E- Dopaminergic phenotype: These PPGLs are characterized by high levels of DA/3-methoxytyramine (3-MT) with normal or near-normal levels of E/MN and NE/NMN (75, 76). Tumors are commonly extra-adrenal and primarily located in the head and neck region (77, 78). Patients can be either asymptomatic or have atypical symptoms like abdominal pain, diarrhea, nausea/vomiting, hypotension, and weight loss. These symptoms are most likely related to dopamine receptor stimulation on the smooth muscle (79), gastrointestinal tract (80), and central nervous system (81). This subset of patients has been traditionally classified as biochemically “silent”. Elevated levels of DA/3MT together with NE have been reported in approximately 65% of patients with *SDHx* mutations, especially in *SDHB* (76, 82).

### Experts recommendations and meta-analysis

The Endocrine Society 2014 guidelines recommend initial screening with either plasma or urine fractionated MN/NMN, with the consensus that values three to four times higher than the upper reference limit are almost always diagnostic for PPGLs (83). A recent meta-analysis done by Därr et al., compared the accuracy of plasma and urine metanephrines in 1,039 patients with PPGL. The study also compared immunoassay methods—HPLC and LC-MS—and differences between supine vs. seated position during sampling. Results in terms of sensitivity/specificity/accuracy showed that supine sampling was more sensitive in tumor detection (95 vs. 89% [*p* < 0.02]). Furthermore, the supine position has a higher specificity than 24-h urine samples (95 vs. 90% [*p* < 0.03]) and the highest accuracy (95%), especially when measured with HPLC and LC-MS over immunoassay (84) as LC-MS eliminates drug interference providing more accurate results.

When evaluating pediatric patients with PPGL, considerations for age-adjusted reference values are crucial to determine the tests positivity. Higher limits for E/MN along with lower limits for NE/NMN were seen in children when compared with adults (85). Finally, we wish to note that LC-MS is not widely available in all countries. Cost of technology itself as well as a lack of trained staff stand as barriers to full implementations of LC-MS (86).

### Imaging

The latest Endocrine Society Guidelines (83) emphasize that consideration for any imaging modality in PPGLs requires prior positive biochemical evidence of disease except for the presence of personal or family history of HNPGL related or not to a hereditary form of the disease. For a general work-up, computed tomography (CT) is recommended as the anatomic imaging modality of choice due to its excellent spatial resolution. Magnetic resonance imaging (MRI) is recommended for children, pregnant women, or patients with HNPGL or metastatic disease. Regarding functional imaging the panel of experts suggests the use of ^123^I-metaiodobenzylguanidine (MIBG) scintigraphy in patients with metastatic disease when treatment with radiotherapy using ^131^I-MIBG is considered or when the risk of metastasis or recurrence of the disease is high based on tumor size. However, if metastatic disease is confirmed, the use of ^18^F-fluorodeoxyglucose (^18^F-FDG) positron emission tomography hybridized with CT (PET/CT) is preferred (83).

We believe PPGL is a disease “born to be filmed” as the availability of high-value modalities for imaging provides a versatile algorithm to evaluate the different scenarios of the disease: in the initial evaluation setting, in treatment planification, and for tumor response assessment. This is due to the expression of various transporters and receptors in the surface of PPGL cells. Receptors that can be exploited for imaging include the NE transporter, glucose transporter (GLUT), amino acid transporter, and somatostatin receptor (SSTR) (87, 88). Currently, there are three types of PET/CT radiopharmaceuticals that exert their actions through these receptors: ^18^F-FDG, ^18^F-fluorodopa (^18^F-FDOPA), and ^68^Galium (^68^Ga)- tetraazacyclododecanetetraacetic acid (DOTA) analogs (89–91). Functional imaging using PET/CT has proven to be superior to ^123^I-MIBG SPECT not only for the higher detection, sensitivity, localization, and resolution, but also for reducing indeterminate or equivocal findings by about 20 to 40% (92). Among these modalities, ^68^Ga- DOTA-Tyr3-octreotate (DOTATATE) PET/CT has emerged as the preferred modality in NETs in general. This is due to the high affinity of the radiolabeled compound for the SSTR type 2 (SSTR_2_) (93) that can more accurately predict a tumor response to treatment to radiolabeled somatostatin analogs in patients with avidity for the tracer. In terms of tumor detection, ^68^Ga-DOTATATE PET/CT has proven to be exceptional with a 98.6% overall detection rate in patients with *SDHB* metastatic PPGL, superior to anatomic imaging (CT/MRI), and other functional scans (^18^F-FDG, ^18^F-FDOPA, and ^18^F-FDA PET/CT) (94). Similar results were also observed in sporadic cases with a sensitivity of 97.6% (89, 95) and in patients with HNPGL (96). Regarding specific mutations, ^68^Ga-DOTATATE PET/CT resulted inferior in the evaluation of patients with polycythemia/PPGL— including both *HIF2A* and *PHD1*-related tumors—(97), *FH* or *MAX* mutations. In these patients— polycythemia associated to PPGL—, the combination of ^18^F-FDOPA PET/CT and ^18^F-FDA PET/CT resulted superior with a lesion-base detection rate of ~98%, vs. 35.3% for the ^68^Ga-DOTATATE PET/CT group (95% CI, 25.0–47.2%) (97). With respect to *FH*-related tumors, in a patient reported ^68^Ga-DOTATATE PET/CT showed an overall detection of 66% when compared to ^18^F-FDOPA PET/CT (98). In reference to *MAX*-related PCCs, in a recent study evaluating six patients, ^68^Ga-DOTATATE accuracy was lower than ^18^F-DOPA PET/CT (99). Based on these studies authors suggest allocating both ^18^F-FDOPA and ^18^F-FDA PET/CT in the diagnostic algorithm of polycythemia/PPGL patients and designate ^18^F-FDOPA PET/CT as first functional imaging modality of choice in the diagnoses and follow-up of both *MAX* and *FH*-related patients. If the availability of ^18^F-FDA and ^18^F-FDOPA PET/CT is limited, use of other imaging modalities like ^123^I-MIBG single-photon emission CT (SPECT) is recommended.

Regarding the pediatric population, ^68^Ga-DOTATATE PET/CT has shown to be superior in a cohort of nine children with *SDHx*-related PPGL with a detection rate of 98.4% when compared to ^18^F-FDG PET/CT (100). However, these results are intriguing as another study reported that the sensitivity of ^68^Ga-DOTATATE PET/CT is lower for abdominal lesions in children, with a detection rate of 66.7% (100, 101), warranting additional larger studies in this population. Thus, the use of more than one functional imaging modality is recommended in the pediatric group and the use of both ^68^Ga-DOTATATE and ^18^F-FDG PET/CT is highly recommended in children with small lesions, when there is a high likelihood of metastatic disease and in those patients with *SDHx* mutations.

The utility of functional imaging using somatostatin analogs, was recently extended and exploited to open new doors for targeted radiotherapy using the both ^177^Lutetium (^177^Lu) or ^90^Yttrium (^90^Y). The ‘so-called' peptide receptor radionuclide therapy (PRRT) has shown to a very effective therapeutic option in patients with advanced midgut NETs in a phase 3 clinical trial called NETTER-1 published in 2017 (102). As a result, ^177^Lu-DOTATATE (Lutathera®) has been recently approved by the food and drug administration (FDA) for the treatment of advanced midgut NETs. Applications of PRRT in PPGL have been assessed also in two small cohorts of patients with mediastinal or HNPGL (103) and with inoperable HNPGL (104), with promising outcomes in terms of tumor response and control of symptoms. A higher number of patients (*N* = 20) received four cycles of Lutathera® with encouraging results in terms of control of symptoms (decreased medication requirements), circulating chromogranin A, tumor response and control of disease with a median progression-free survival (PFS) of 29 months (105). When comparing PRRT with ^131^I-MIBG in 22 patients with metastatic/progressive PPGL, PRRT showed increased PFS and tumor response rate, as well as increased event-free and overall survival (OS) (106). An ongoing prospective clinical trial at the NIH evaluating PRRT for progressive PPGL will provide definite answers regarding the utility and safety of PRRT in PPGL (NCT03206060).

The role of SSTR antagonists appears to be promising in the field of NETs. SSTR antagonists recognie more binding sites on SSTRs allowing better tumor visualization (107). Their clinical utility in functional imaging was first demonstrated in 2011 (108). A second-generation of SSTR_2_ antagonists that include DOTA-JR11 showed higher tumor uptake when combined with ^68^Ga-DOTA (1.3 times), or ^68^Ga-NODAGA (1.7 times) as compared with DOTA analogs (109). In the preclinical setting, DOTA-JR11 was superior to ^177^Lu in H69 cell lines, and *in vivo* therapy experiments achieved a higher uptake, median survival rate, and a longer delay in tumor growth (110). Regarding pharmacokinetics and dosimetry, DOTA-JR11 showed very promising results when tested in human embryonic kidney cells with a higher uptake and longer tumor residence time. With an escalating dose DOTA-JR11 demonstrated an improved safety profile and the potential decrease toxicity (111). In *in vitro* studies including both NETs and non-NETs, DOTA-JR11 showed a higher affinity for SSTTRs when compared to the SSTR_2_ agonist ^125^I-Tyr3-octreotide in NETs (2.5 to 40 times). Interestingly, the group of other non-NETs tumors— such as breast cancer, renal cell carcinoma, medullary thyroid cancer, and non-Hodgkin lymphoma—, was also targeted with high affinity, potentially opening a new door for exploring this modality in tumors where typically SSTR have played little role, if any (112). Currently, an ongoing clinical trial for metastatic and unresectable progressive, well-differentiated carcinoid is trying to elucidate the role of SSTR antagonist DOTA-JR11 both in diagnostic and therapeutic settings (NCT02609737).

## Conclusion

**Figure 3:**
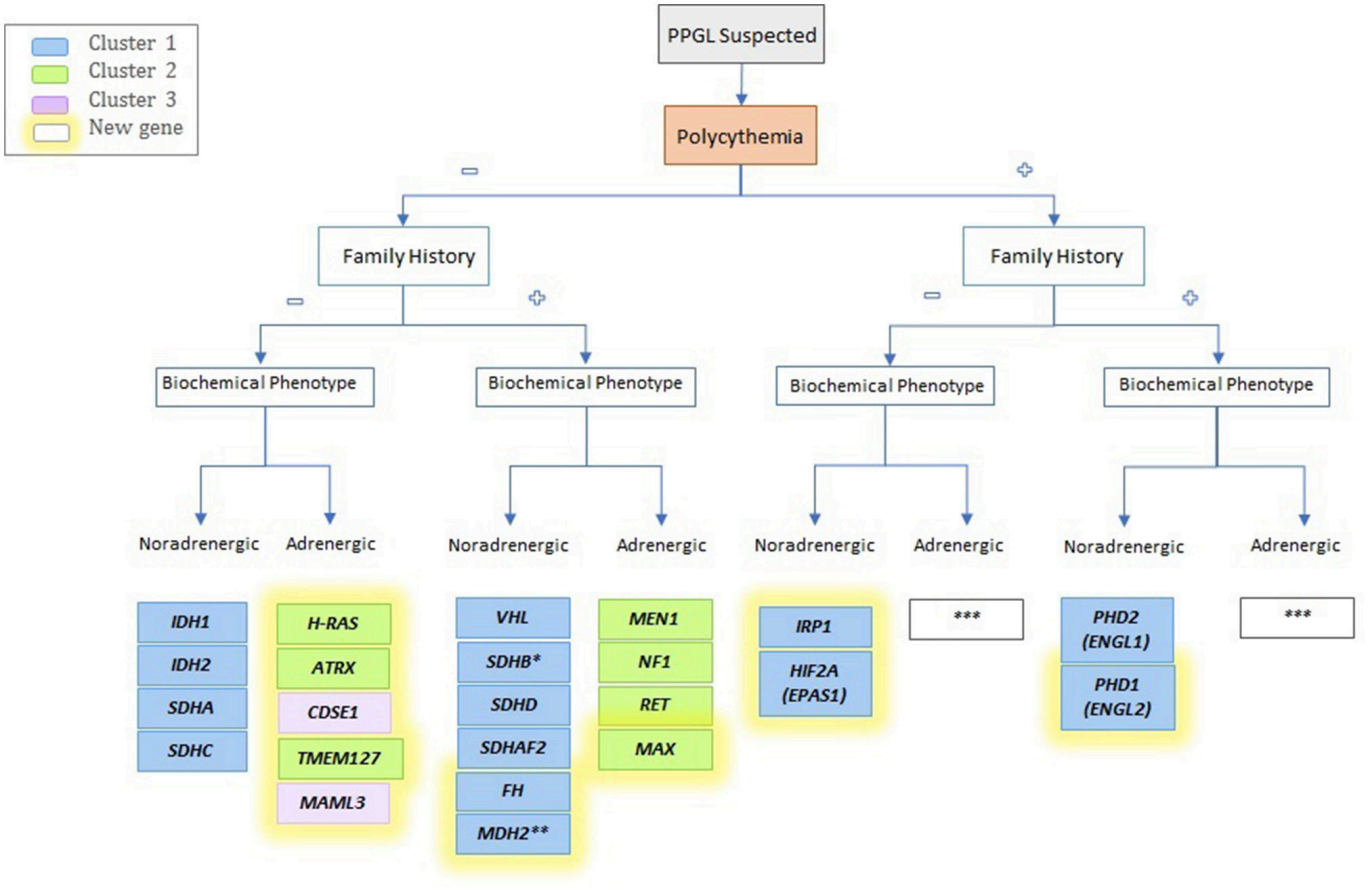
Schematic algorithm for identifying a possible driver mutation of the newly identified genes mutation. *Often family history is absent; **Unknown; ***Not described yet.

In this article, we presented some of the latest advances in the rapidly evolving field of PPGL and we focused on genetic, biochemical and imaging discoveries over the last 6 years. In addition, we proposed an updated biochemical classification and provided a novel algorithm for identifying newly diagnosed PPGL (Figure 3). Ten new susceptibility genes related to PPGL have been described in the last 10 years, including new germline as well as somatic mutations. The last can be presented as mosaicisms and result in syndromic forms of the disease. Also, functional imaging modalities continue to improve, and lesion-base detection studies are more reliable and accurate. The wide availability of ^68^Ga-DOTATATE PET/CT following the approval of the radiopharmaceutical compound by the FDA, is revolutionizing the way we diagnose not only PPGLs but also NETs in general. The expression of SSTR^2^ on PPGL cells has allowed the exploitation of this modality as a therapeutic option in these patients using Lutathera®, which was recently approved for use in midgut NETs. Current ongoing clinical trials in PPGL will determine safety, tolerability profile, and also efficacy in terms of clinical benefit and control of disease. Hopefully the availability of these diagnostic modalities will be more implemented in developing countries in the future. The expense associated with these technologies stands as a barrier; therefore, referral to centers of excellence that specialize in PPGL is warranted and highly advisable as implementation of theranostics into an algorithm is required for establishing different therapeutic options.

We are now entering a new and exciting era for PPGL that will allow us to advance one step further toward personalized medicine, making precision medicine for PPGL a step closer (Figure 2).

## Author contributions

RA: writing and editing MS, creating Table 1 and Figure 3, proposing the idea of an update in the biochemical classification; AS: writing MS; IT: writing, editing and reviewing MS, creating Figures 1, 2; KP: creating outline and reviewing MS.

### Conflict of interest statement

The authors declare that the research was conducted in the absence of any commercial or financial relationships that could be construed as a potential conflict of interest.

## Abbreviations

3-MT: 3-methoxytyramine
^18^FDA: ^18^F-fluorodopamine
^18^F-DOPA: ^18^F-fluorodopa
^18^F-FDG: 18F-fluorodeoxyglucose
^68^Ga: ^68^Galium
^90^Y: ^90^Ytrium
^131^I-MIBG, ^131^I-MIBG: metaiodobenzylguanidine
^111^In: ^111^Indium
^177^Lu: ^177^Lutethium
AFAP1: apoptosis protease activator protein 1
AKT: serine/threonine kinase
ATRX: chromatin remodeler ATRX
CT: computerized tomography
CRG: growth regulatory factors
CSDE1: cold-shock domain containing E1
CVD, cyclophosphamide: vincristine and dacarbazine
DA: dopamine
DIPG: diffuse intrinsic pontine glioma
DOTA: tetraazacyclododecanetetraacetic acid
DOTATATE: DOTA-Tyr3-octreotate
DS: direct sequencing
E: epinephrine
EGLN1/2: egl nine homolog 1 and 2
EPAS1: PAS domain-containing protein 1
EPO: erythropoietin
EpoR: erythropoietin receptor
ERK: extracellular mitogen-activated protein kinase 1
FDA: food and drug administration
FH: fumarate hydratase
HNPGL: head and neck paraganglioma
GTC: giant cell tumor of the bone
GTP: guanosine-5'-triphosphate
H3F3A: H3 histone family member 3A
NHPGL: head and neck PGL
HIF2α: hypoxia-inducible factor 2 alpha
HIF2A: hypoxia-inducible factor 2 alpha
HLPC: high-performance liquid chromatography
HLRCC: leiomyomatosis and renal cell cancer
H-RAS: HRAS proto-oncogene
IDH1/2: isocitrate dehydrogenase 1 and 2
IHC: immunohistochemistry
IRP1: iron regulatory protein
MCUL: multiple cutaneous leiomyomatosis
MDH1/2: malate dehydrogenase type 1 and 2
MAML3: coactivator 3 mastermind-like
MAPK: mitogen-activated protein kinase
MAX: myc-associated factor X gene
Men1: multiple endocrine neoplasia 1
LC-MS: liquid chromatography tandem-mass spectrometry
MEK: mitogen-activated protein kinase
MLPA: multiplex ligation-dependent probe amplification
MN: metanephrine
MRI: magnetic resonance imaging
mRNA: messenger ribonucleic acid
mTOR: mammalian target of rapamycin
NE: norepinephrine (NE)
NETs: neuroendocrine tumors
NF1: neurofibromin 1
NIH: National Institutes of Health
NMN: normetanephrine
NET: neuroendocrine tumor
PCC: pheochromocytoma
PET: positron emission tomography
PGLs: paraganglioma
PHD1/2: prolyl hydroxylase 1 and 2
PI3K: phosphatidyl-inositol-3-kinase
PPGL: pheochromocytoma-paraganglioma
PRRT: peptide receptor radionuclide therapy
RF: radiofrequency
SDH: succinate dehydrogenase subunits A/B/C/D
SDHAF2: succinate dehydrogenase complex assembly factor 2
SPECT: single photon emission computed tomography
SSA: somatostatin analogs
SSTR: somatostatin receptor
TCA, tricarboxylic acid, TA: thermoablation
TCGA, The Cancer Genomic Atlas, TFG: transcription factors genes
TMEM127: transmembrane protein 127
TMZ: temozolomide
TSG: tumor suppressor gene
UBTF: upstream binding transcription factor
VHL: von Hippel Lindau
WES: whole exome sequencing
WHO: World Health Organization.
